# Advancements in Inflammatory Bowel Disease Management: From Traditional Treatments to Monoclonal Antibodies and Future Drug Delivery Systems

**DOI:** 10.3390/pharmaceutics16091185

**Published:** 2024-09-07

**Authors:** Annalisa Di Rienzo, Lisa Marinelli, Marilisa Pia Dimmito, Eleonora Chiara Toto, Antonio Di Stefano, Ivana Cacciatore

**Affiliations:** Department of Pharmacy, “G. d’Annunzio” University of Chieti-Pescara, 66100 Chieti, Italy; annalisa.dirienzo@unich.it (A.D.R.); marilisa.dimmito@unich.it (M.P.D.); eleonora.toto@unich.it (E.C.T.); antonio.distefano@unich.it (A.D.S.); ivana.cacciatore@unich.it (I.C.)

**Keywords:** Crohn’s disease, gastrointestinal inflammation, inflammatory bowel disease, monoclonal antibodies, ulcerative colitis

## Abstract

Inflammatory bowel disease (IBD) is a chronic gastrointestinal inflammatory disorder with two main subtypes: ulcerative colitis (UC) and Crohn’s disease (CD). The pathogenesis involves genetic predisposition, dysbiosis, and immune dysregulation. Complications include perianal lesions, strictures, fistulas, perforations, and an increased risk of colon cancer. Clinical classification ranges from mild to fulminant and recurrent disease, with common symptoms such as abdominal discomfort, rectal bleeding, diarrhea, and weight loss. Extraintestinal manifestations include arthritis, erythema nodosum, pyoderma gangrenosum, and uveitis. Conventional treatments using aminosalicylates, corticosteroids, and immunomodulators have limitations. Biologics, introduced in the 1990s, offer improved efficacy and specificity, targeting factors like TNF-α, integrins, and cytokines. Monoclonal antibodies play a crucial role in IBD management, aiming to reduce relapses, hospitalizations, and surgeries. In conclusion, this review is aimed at summarizing the latest knowledge, advantages, and drawbacks of IBD therapies, such as small molecules, biologics, and monoclonal antibodies, to provide a basis for further research in the IBD field.

## 1. Introduction

Inflammatory bowel diseases (IBDs) represent a class of chronic inflammatory disorders of the gastrointestinal system, characterized by constant relapsing and remitting events. It comprises two main subtypes: ulcerative colitis (UC) and Crohn’s disease (CD). The pathogenesis of these diseases is not completely known. Genetic predisposition, dysbiosis (the alterations of the gut microbiota barrier), the dysregulation of immune responses (innate and adaptive), and environmental factors could represent the main causes of this inflammation. The incidence of IBD is higher for men than women in UC, but in CD the incidence is equal [1]. CD affects the gastrointestinal tract, from mouth to the anus, causing transmural inflammation while UC inflammation is restricted to the colon and rectum. The major complications of IBD are represented by the development of perianal skin lesions or anal canal lesions such as crypt abscesses, fistulas, strictures, and also perforations, stenosis or gastrointestinal bleeding, and progressive bowel damage that demands essential surgery [2,3,4]. 

According to the clinical classification, CD as well as UC can be subdivided into mild, moderate or severe, and fulminant disease. Common symptoms are abdominal discomfort, rectal bleeding, diarrhea, tenesmus, weight loss, and anemia. Colon cancer, more frequent in UC, corresponds to the worst scenario. IBD occurs not only through intestinal damage, but also extraintestinal manifestations can arise, including arthritis, erythema nodosum, pyoderma gangrenosum, episcleritis, and uveitis [5].

Conventional treatments for IBD consisting of aminosalicylates, corticosteroids, and immunomodulators are the first-line therapy. These drugs were introduced in the 1950s, showing good properties such as low molecular weight, optimal stability, low costs, and oral administration. However, side effects occur causing the loss of response and refractory to the treatment by patients, proving the introduction of new treatments necessary [6,7]. Notably, biologics were introduced in the 1990s to ameliorate the quality of patients’ lives, lowering relapses, hospitalization, and surgery. These therapies are based on monoclonal antibodies characterized by a long half-life and more effectiveness and specificity than traditional drugs. These monoclonal antibodies target important factors that are activated in the inflammatory process, such as Tumor Necrosis Factor-α (TNF-α), integrins, and cytokines [8,9]. Moreover, Janus kinase (JAK) inhibitions emerge as an alternative strategy in IBD. They are small molecules able to modulate downstream cytokine signaling in immune-mediated diseases, also involved in the pathogenesis of IBD. In more detail, tofacitinib, upadacitinib, and filgotinib are examples of the approved drugs for ulcerative colitis (UC) in patients not responding to conventional therapy or biological drugs. Compared to biological drugs, these small molecules possess many advantages including a lack of immunogenicity, suitability for oral administration, and low manufacturing cost [10]. 

Due to the importance and relevance of IBD as a worldwide disease, herein, we focused the attention on the gut structure changes and on the main biological strategies approved for managing IBD or achieving remission, their mechanisms of action, and future prospects in this field.

The data collected for this review were derived from “Pubmed”, “Scopus”, and regulatory databases by searching the following keywords: inflammatory bowel diseases, traditional treatments, monoclonal antibodies, drug delivery systems, aminosalicylates, corticosteroids, immunomodulators, Tumor Necrosis Factor-α (TNF-α) blockers, integrin blockers, interleukin blockers, infliximab, adalimumab, golimumab, certolizumab pegol, natalizumab, vedolizumab, etrolizumab, ustekinumab, mirikizumab, briakinumab, ontamalimab, risankizumab, brazikumab, and guselkumab. On the 25th of July 2024, the research was completed.

## 2. Gut Barrier in IBD

The intestinal epithelial barrier has a pivotal role in maintaining the gut homeostasis and to guarantee the defense of the mucosal immune system [11]. It is covered by a thick mucus layer, composed of mucin (Muc2, secreted by goblet cells) and formed by heterogeneous cells, like enterocytes, goblet cells, neuroendocrine, Paneth, and M cells, and enteroendocrine cells, interconnected by tight junctions. It is proved that the deletion of Muc2 causes the formation of spontaneous colitis [12,13,14]. Immunoglobulin A (IgA) contrasts the invasion of pathogens and maintains the homeostasis between the host and commensal microorganisms. However, the deficiency of IgA alters the permeability of the gut barrier. Commensal bacteria located in the lumen are separated from the lamina propria by this barrier [15]. Pattern-recognition receptors (PRRs) are responsible for bacteria recognition and determine the initiation of immune response. The membrane tool-like receptors (TLRs) and the intracellular nucleotide-binding oligomerization domain (NOD)-like receptors (NLRs) are the main classes of PRRs. These PRRs allow us to discern pathogen-associated molecular patterns (PAMPs) and damage-associated molecular patterns (DAMPs). Commensal microbiota binds PRRs on the apical membrane due to the increasing barrier permeability and the subsequent disruption of tight junctions stimulates the activation of the macrophages and dendritic cells belonging to the innate immune system [16]. The lack of membrane integrity triggers the activation of innate inflammatory response [17,18]. Antigen-presenting cells (APCs), like macrophages and dendritic cells, recognize the host pathogens and activate the nuclear factor-kB (NF-kB) pathways, stimulating the transcription of pro-inflammatory genes, increasing the production of pro-inflammatory cytokines, especially TNF-α and interleukins, such as IL-12 and IL-23, released by macrophages and dendritic cells after presenting antigens. These interleukins present receptors on naïve CD4+ T cells, stimulating and activating the adaptative immune system and promoting differentiation into Th1, Th2, and Th17. The Th1 pathway is predominant in CD, while Th2 and Th17 are in UC. In addition, T cells are moved from the lamina propria to the blood vessels due to the interaction between α4β7 integrin with mucosal addressing cell adhesion molecule-1 (MAdCAM-1), increasing the amount of gut-specific T cells into the lamina propria [11]. Furthermore, the elevated production of TNF-α induces the necrosis of Paneth cells, activation of macrophages, and damage of gut cells, causing the chronic inflammation of the intestine [19,20]. 

## 3. Traditional Treatments of IBD

### 3.1. Aminosalicylates

The first line of therapy, commonly used for the healing of IBD, is based on the traditional drugs administered as suppositories, enemas, foams, tablets, or granules. The first aminosalicylate employed in the treatment of IBD was sulfasalazine, a prodrug composed of 5-aminosalicylic acid (known as mesalamine) and sulfapyridine linked by a diazo binding [21]. After oral administration, it reaches the colon and releases 5-aminosalicylic acid, the active drug, and sulfapyridine that is metabolized. Aminosalicylates exert their action by inhibiting IL-1 and TNF-α production and the NF-kB and lipoxygenase pathways. Sulfasalazine is indicated for patients affected by mild or moderately active UC. The optimal dose is 2–2.4 g/day; however, in the case of more severe colitis, combined therapy with glucocorticoids is advised [22]. The main side effects such as infertility, hemolytic anemia, hepatitis, rash, and fever, are related to the sulfa moiety. Moreover, sulfasalazine can cross the placenta while mesalamine can cause interstitial nephritis [23]. To improve these aspects, new derivatives were designed using other 5-aminosalicylic acid or inert drugs, such as olsalazine and balsalazide [24,25]. These drugs are orally administered, and they are indicated for improving the remission rate in CD and UC [26]. 

### 3.2. Corticosteroids

Alternative drugs for patients who have not responded to mesalamine within 2–4 weeks with mild to moderate CD and severe UC are corticosteroids [7,27,28]. They are anti-inflammatories used for IBD since the 1950s. Notably, the first generation of corticosteroids, consisting of hydrocortisone, methylprednisolone, and prednisone, represents a valid treatment, either alone or in combination with mesalamine. Although prednisone, administered orally or parenterally for emergency, showed good efficacy, benefits terminate after 9 months. The initial dose of prednisone is 40–60 mg/day until the gradual reduction to control symptoms. However, several side effects are related to the first generation of corticosteroids, such as osteoporosis, hypertension, ocular effects, and diabetes mellitus, and limit their use as a maintenance therapy. Due to the limited time of the treatment with first-generation therapy, budesonide and beclomethasone dipropionate have been developed as second-generation [29]. These drugs, orally or topically administered, can minimize side effects through the first-pass metabolism that causes the inactivation of derivatives. Oral beclomethasone dipropionate has been approved for UC [30]. 

### 3.3. Immunomodulators

In steroid-resistant or steroid-dependent patients, immunomodulators, like thiopurines, methotrexate, and calcineurin inhibitors, can be a valid option. Thiopurines consisting of azathioprine, 6-mercaptopurine, and 6-thioguanine exert their action hampering T lymphocyte proliferation and activation [31]. Thiopurines can be considered as the first line of preventing therapy for patients after postoperative events with a risk of relapse. Purines are used only in maintenance due to their efficacy being reached after 3 months. Besides, methotrexate is also for induction and maintenance. Azathioprine is a prodrug, converted into mercaptopurine, and metabolized into the active drug 6-thioguanine nucleotides. Azathioprine is usually given at 2–2.5 mg/kg while mercaptopurine is administered at doses of 1.5 mg/kg. The development of pancreatitis, lymphoma, or leukemia can be connected to azathioprine administration [32]. 

Methotrexate is revealed to be more beneficial and effective than thiopurines. It is administered parenterally and used only for the maintenance of medical remission in CD [33]. It is well known as an inhibitor of dihydrofolate reductase, leading consequently to cell death; moreover, it possesses anti-inflammatory properties, lowering pro-inflammatory cytokine production and leading to the apoptosis of T cells [34]. It is safe and tolerable and can be given subcutaneously. However, it is considered teratogenic hampering administration in patients during pregnancy. The main adverse effect of long-term therapy is represented by hepatic fibrosis. Cyclosporine and tacrolimus are calcineurin inhibitors, widely used in organ transplantation [35,36]. Calcineurin, after its activation by calmodulin, dephosphorylates and activates the nuclear factor of activated T cells, which regulates the gene transcription of pro-inflammatory cytokines. Both compounds bind to calcineurin and inhibit the transcription of T cells, causing the down-regulation of the production of pro-inflammatory cytokines, especially IL-12, IL-23, and TNF. Cyclosporine can be given orally but, due to the first-pass effect, it is more potent intravenously, allowing one to avoid surgery. Nevertheless, it has several side effects, especially renal insufficiency, and hypertension. Tacrolimus is a macrolide antibiotic like cyclosporin, better absorbed orally; it can also be administered intravenously in the case of severe UC [37]. The main characteristics of the traditional drugs are reported in Table 1.

## 4. Monoclonal Antibodies in IBD

**Figure 1:**
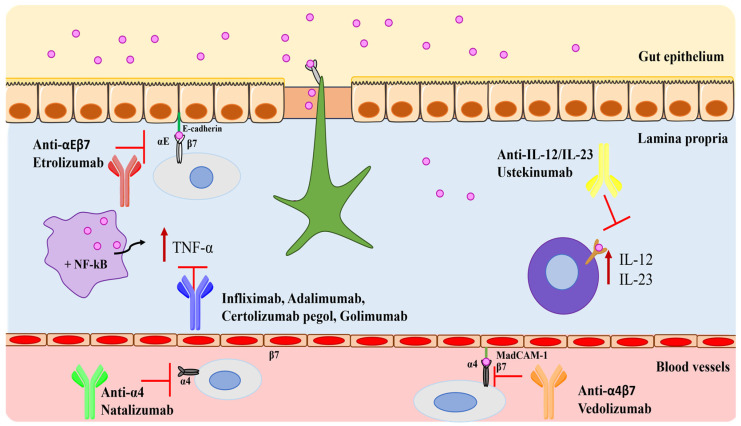
Main targets of biological drugs in the treatment of IBD.

The advent of biological therapies has revolutionized the conventional therapies of IBD, exploiting a new strategy of action against selective inflammation pathways [38,39]. These therapies, summarized in Table 2, are based on the monoclonal antibodies able to target and inhibit TNF-α, integrins, and the principal cytokines involved in the inflammatory processes (Figure 1). The use of these therapies is preferred because they can act directly on the main process of the disease and improve the clinical remission and maintenance of relapsing [40,41]. 

### 4.1. Tumor Necrosis Factor-Alpha (TNF-α) Blockers

TNF plays a crucial role as a driver of inflammatory pathways in the gut [20]. It exists in two forms: transmembrane (tmTNF) and soluble (sTNF). The tmTNF is a precursor form consisting of 26 kDa subunits. After cleavage by the TNF-α-converting enzyme (TACE), it is converted into the sTNF, a 17 kDa non-glycosylated protein. TNF is relevant in the activation of immune response after binding to two receptors, TNFR1 and TNFR2. TNFR1 is ubiquitously expressed in almost all cell types, while TNFR2 is only located in lymphocytes and endothelial cells. Due to the linkage to the receptor, it causes the production of cytokines, such as IL-1β and IL-6, and interferon-γ, as well as the activation of macrophages and T cells [19,42]. TNF-α blockers are the first biologics used for the treatment of moderate to severe CD and UC as pivotal resources to improve the induction and maintenance of remission and to reduce relapses, surgery, and hospitalizations. The action of these inhibitors does not regard only the block of TNF-α but also the induction of the T cell apoptosis [43].

Infliximab is a chimeric antibody, containing 75% human and 25% murine IgG1 discovered in 1998 and it was the first biologic approved by the Food and Drug Administration in 1998 and 2006, for CD and UC, respectively, with the commercial name of Remicade^®^ [44,45]. Like other biologics, it is approved also as a treatment for rheumatoid arthritis, ankylosing spondylitis, psoriasis, and psoriatic arthritis [46]. It exerts its mechanism of action inhibiting the binding between soluble and transmembrane TNF-α and its receptor, hampering the initiation of inflammatory pathways and the production of cytokines such as IL-1 and IL-6. It is administered intravenously at the recommended dose of 5 mg/Kg starting from a phase of induction dose of 0, 2, and 6 weeks followed by a maintenance treatment every 8 weeks with a half-life of eight days [46,47]. The risk of administering infliximab is represented by a loss of response and efficacy attributed to the low active drug available, which may be caused by the formation of antidrug antibodies (ADAs) (hypersensitivity reactions) [48,49]. Furthermore, infliximab can cross the placenta in the case in which mothers take infliximab during pregnancy for up to six months. Many reactions can be presented in these babies after administering live vaccines; for this reason, it should be advised to wait six or more months before administering vaccines to the infants. The side effects known are infusion reactions, a loss of response, and hypersensitivity [50,51]. Infliximab is indicated for the treatment of moderate to severe UC and CD in adults who have had a good response to traditional therapy or who are intolerant to or have highlighted side effects for specific therapies. Currently, infliximab is preferred because its intravenous route is able to have a faster distribution than the other anti-TNF-α [52,53]. The FDA in October 2023 approved the first subcutaneous (sc) formulation of infliximab, infliximab-dyyb (Zymfentra^®^), for the maintenance treatment of IBD [54].

Adalimumab (Humira^®^) is a recombinant human IgG1, approved after infliximab for IBD in 2003 [55,56]. It is administered intramuscularly and has a half-life of 10–13 days, longer than infliximab [57]. Prevalent adverse events are injection site reactions, respiratory infections, headaches, and nausea. It blocks TNF-α and causes alterations in the levels of cellular adhesion to activate leukocyte migration [57].

Golimumab (Simponi^®^) was approved for the treatment of moderate to severe UC by the FDA and EMA in 2013 [58]. It was approved and mainly used for rheumatoid arthritis, ankylosing spondylitis, and psoriatic arthritis [59]. Golimumab is a recombinant antibody, human IgG1, with a 2-week half-life, which acts inhibiting both forms of TNF-α, administered via the subcutaneous route in pre-filled syringes as alternatives to conventional drugs or ineffective biologics [60]. 

Certolizumab pegol (Cimzia^®^) is a particular humanized antibody composed of a polyethylene glycolate Fab fragment linked to a 40 kDa polyethylene glycol molecule (PEG) and free of the crystallizable fragment (Fc) [61,62]. Certolizumab is approved for CD only in the USA, Switzerland, and Russia, but also for rheumatoid arthritis, psoriatic arthritis, ankylosing spondylitis, plaque psoriasis, and non-radiographic spondyloarthritis [63]. It is administered subcutaneously at weeks 0, 2, and 4 for the induction of remission and then every 4 weeks for the maintenance of remission. It has a longer half-life than the other anti-TNF thanks to the presence of PEG [64].

### 4.2. Integrin Blockers

Integrins are cell surface glycoproteins involved in the interaction with cell adhesion molecules (CAMs) expressed on other cells through the two subunits α and β [65]. This binding has a key role in the mediation of the trafficking and retention of immune cells in the gut lumen. Indeed, lymphocyte migration can be driven by the integrin α4β1 (VLA-4) and vascular cell adhesion molecule 1 (VCAM-1) [66]. Homing lymphocytes from the blood to the gut lumen can be favored by α4β7 integrin able to bind MAdCAM-1. Furthermore, the integrin αEβ7 (also known as CD103) interacts with E-cadherin and leads to the retention of lymphocytes at the gut layer. The up-regulation of VCAM-1 and MAdCAM-1 is a possible cause of IBD due to the increase in the circulation of lymphocytes in the gut. For these reasons, several integrin inhibitors are currently used in IBD treatment [67,68].

Natalizumab (Tysabri^®^), approved for CD in Switzerland, is a humanized monoclonal IgG4 antibody that is selective for binding to the α4 subunit, interrupting the interactions between the α4β7 and MAdCAM-1 as well as α4β1 and VCAM-1 [69]. It is intravenously administered, resulting effectively in those patients affected by moderately to severely active CD [69]. However, its use is associated with many cases of progressive multifocal leukoencephalopathy (PML) related to the John Cunningham (JC) virus [70,71].

Vedolizumab (Entyvio^®^) is a humanized monoclonal IgG1 antibody blocking the bond between the heterodimer α4β7 integrin and the MAdCAM-1. It was approved by the FDA in 2014 for the induction and remission of CD and UC [72]. The route of administration is intravenous, and it was not associated with an increased risk of severe or opportunistic infections, including sepsis, tuberculosis, *Listeria meningitis*, and clostridial infections, showing a safe profile [73,74].

Etrolizumab is a humanized monoclonal IgG1 antibody that acts by targeting the β7 subunit of both α4β7 and αEβ7 integrins. Etrolizumab binds α4β7 and αEβ7, avoiding the binding to MAdCAM-1 and E-cadherin, respectively, blocking the trafficking of lymphocytes to inflammatory sites at the gut level. These gut-specific lymphocytes are factors that contribute to an inflammatory process in UC and CD [75].

### 4.3. Interleukins Blockers

Interleukin IL-12 and IL-23 are heterodimeric cytokines consisting of the subunits p40 and p35 for IL-12 and the subunits p40 and p19 for IL-23 [76,77]. They are released and secreted by macrophages and dendritic cells. Their receptors are expressed on the surface of natural killer cells and T cells. Notably, IL-12 binds through the p40 subunit to the IL-12 receptor (IL-12R) β1 and IL-12Rβ2 chains presented on the surface of T cells or natural killer cells. The activation of this receptor starts the signal transduction of transcription STAT4 and STAT6 proteins, NK lysis, and the production of cytokines, such as IFN-γ and TNF-α. IL-12 has the role of stimulating natural killers and the differentiation of CD4+ T cells towards Th1, while IL-23 the Th17. IL-23 binds to its specific receptor, IL-23R, activating Janus kinases JAK-2 and Tyk-2, which activate the nuclear translocation of the transcription factors STAT3 and STAT. Moreover, it exerts the production of innate lymphoid cells type 3, granulocytes and natural killer cells, and pro-inflammatory cytokines [78]. It is known that CD and UC are caused by an abnormal regulation of these interleukins. Starting from this evidence, interleukin blockers composed of monoclonal antibodies have been developed.

Ustekinumab (Stelara^®^) is a monoclonal IgG1 kappa, indicated for the treatment of adult patients with moderate to severe CD or UC. It is indicated in the case of the absence of responses, intolerants, and side effects of traditional drugs or TNF-α antagonists. It is approved for the care of psoriasis, psoriatic arthritis, CD (2016), and recently for UC (2019) [79]. It is administered intravenously the first time and then after 8 weeks subcutaneously through injection in pre-filled syringes or pens. The administration for maintenance is recommended every 12 weeks. Ustekinumab exerts its mechanism of action by binding specifically to the shared p40 protein subunit of IL-12 and IL-23, interrupting the linkage with the IL-12Rβ1 receptor and, in this way, the activation of the inflammatory pathway. The half-life is close to 3 weeks. Reactions common to the other monoclonal antibodies, like tuberculosis and hypersensitivity, can be manifested [80,81]. Mirikizumab (Omvoh^®^) is a selective IL-23p19 antagonist administered for the treatment of moderate to severe UC approved by EMA in 2023 [82,83]. 

### 4.4. Monoclonal Antibodies in Development

Several studies regarding novel targets involved in the pathogenesis of IBD are still ongoing to create an alternative to the current treatments. Briakinumab (ABT-874) is a human monoclonal antibody able to inhibit the p40 subunit of IL-12 and IL-23. Currently, it has not been approved by the FDA for the treatment of CD or UC [84]. These are focused on the direct binding of the monoclonal antibody to MAdCAM-1, to the subunit p19 of IL-23, and many studies also are studying the IL-23/IL-17 axis. PF-00547659, a monoclonal IgG2 antibody, and ontamalimab, a fully human mAb characterized by good tolerability in the long term and an optimal safe profile, bind selectively to MAdCAM-1, blocking the bind with the α4β7 integrin [85]. Biologics that target the subunit p19 of the interleukin 23 are risankizumab, a humanized IgG1 monoclonal antibody, and brazikumab [86]. Recently, the IL-23/IL-17 axis has attracted much attention, especially in severe UC, due to its ability to promote Th17 cells and cytokine-related immune response. IL17 is a large family composed of six proteins. Its activation sustains the release of pro-inflammatory pathways and mediators. However, trials regarding biologicals that inhibit this target are currently under investigation. The IL-23/IL-17 axis blockage could be an alternative strategy in the treatment of IBD [87]. Guselkumab is a fully human immunoglobulin G1 lambda (IgG1λ) monoclonal antibody targeting with high affinity and specificity the p19 subunit of human IL-23. Guselkumab is approved for moderate to severe plaque psoriasis and active psoriatic arthritis. Moreover, clinical trials on patients with moderate to severe CD showed good results, also revealing a good safety profile [88]. 

**Table 2:** Summary of monoclonal antibodies for the treatment of IBD.

Drugs	Mechanism of Action	Administration Route	Doses	Adverse Effects	Ref.
**Infliximab** **(REMICADE^®^)**	Anti-TNF-α	Intravenous	5 mg/kg Induction doses: 0, 2, and 6 weeks. Maintenance doses: 8-weekly intervals.	Hypersensitivity reactions.	[44]
**Infliximab** **(ZYMFENTRA^®^)**	Anti-TNF-α	Subcutaneous	Maintenance doses only: 120 mg at week 10, 2-weekly intervals.	COVID-19, anemia, arthralgia, injection site reaction, increased alanine aminotransferase, and abdominal pain (for UC). COVID-19, headache, upper respiratory tract infection, injection site reaction, diarrhea, increased blood creatine phosphokinase, arthralgia, increased alanine aminotransferase, hypertension, urinary tract infection, neutropenia, dizziness, and leukopenia (for CD).	[54]
**Adalimumab** **(HUMIRA^®^)**	Anti-TNF-α	Intramuscular	Induction doses: 80/160 mg at week 0 and 40/80 mg at week 2 (for CD); 80/160 mg at week 0, 40/80 mg at week 1, and 40/80 mg at week 2 (for UC). Maintenance doses: 20/40 mg every week starting from week 4 (for CD); 40/80 mg every week starting from week 4 or 20/40 mg every week (for UC).	Infections (e.g., upper respiratory, sinusitis), injection site reactions, headache, and rash.	[55]
**Golimumab** **(SIMPONI^®^)**	Anti-TNF-α	Subcutaneous	Induction doses: 200 mg week 0 and 100 mg at week 2. Maintenance doses: 50/100 mg 4-weekly intervals.	Paraesthesia, cutaneous infection, pneumonitis, and the recurrence of cervical neoplasia.	[58]
**Certolizumab pegol** **(CIMZIA^®^)**	Anti-TNF-α	Subcutaneous	Induction doses: 400 mg at 0, 2, and 4 weeks. Maintenance doses: 400 mg 4-weekly intervals.	Upper respiratory tract infection, rash, and urinary tract infection.	[61]
**Natalizumab** **(TYSABRI^®^)**	Integrin blocker	Intravenous	300 mg 4-weekly intervals.	Headache, the exacerbation of CD, nausea, nasopharyngitis, and progressive multifocal leukoencephalopathy.	[69,70]
**Vedolizumab** **(ENTYVIO^®^)**	Integrin blocker	Intravenous and subcutaneous	Induction doses: 300 mg at weeks 0, 2, and 6. Maintenance doses: 300 mg 8-weekly intervals.	Nasopharyngitis, headache, arthralgia, nausea, pyrexia, upper respiratory tract infection, fatigue, cough, bronchitis, rash, pruritus, sinusitis, oropharyngeal pain, pain in extremities, and injection site reactions with subcutaneous administration.	[72,73]
**Etrolizumab**	Integrin blocker	Subcutaneous	105 mg every 4 weeks for 14 weeks (phase 3 clinical program).	UC flare, appendicitis, and anemia.	[75]
**Ustekinumab** **(STELARA^®^)**	Interleukin blocker	Intravenous and subcutaneous	Induction doses: depends on the patient’s body weight. Maintenance doses: 90 mg 8–12-weekly intervals.	Headache, nasopharyngitis, inflammation of the nose and throat, and hypersensitivity (allergic reaction).	[79]
**Mirikizumab** **(OMVOH^®^)**	Interleukin blocker	Intravenous and subcutaneous	Induction doses: 300 mg at weeks 0, 4, and 8; 4-weekly intervals. Maintenance doses: 200 mg 4-weekly intervals.	Infections (active tuberculosis).	[83]
**Briakinumab**	Interleukin blocker	Intravenous and subcutaneous	Induction doses: 200, 400, and 700 mg at weeks 0, 4, and 8. Maintenance doses: 200, 400, and 700 mg after 12 weeks (phase 2 clinical program).	Upper respiratory tract infection, nausea, abdominal pain, and headache.	[84]
**Ontamalimab**	Integrin blocker	Subcutaneous	Induction doses: 25/75 mg once 4-weekly intervals to week 12. Maintenance doses: 25/75 mg once at 4-weekly intervals to week 52 (phase 3 clinical program).	Serious infections, gastroenteritis, pelvic abscess, pneumonia, arthralgia, and nasopharyngitis.	[85]
**Risankizumab** **(SKYRIZI^®^)**	Interleukin blocker	Intravenous and subcutaneous	Induction doses: 600 mg at weeks 0, 4, and 8. Maintenance doses: 360 mg at week 12, 8-weekly intervals.	Upper respiratory infection, tinea infection, folliculitis, headache, pruritus, rash, urticaria, fatigue, and injection site reactions.	[86]
**Brazikumab**	Interleukin blocker	Intravenous and subcutaneous	Induction doses: 700 mg at weeks 0 and 4. Maintenance doses: 210 mg from week 12. (Phase 2 clinical program.)	Nasopharyngitis, headache, and abdominal pain.	[86]
**Guselkumab** **(TREMFYA^®^)**	Interleukin blocker	Intravenous	Induction doses: 200 mg at weeks 0, 4, and 8 for 12 weeks.	Headache, joint pain, upper respiratory infections, diarrhea, and stomach pain.	[88]

### 4.5. Other Treatments

Since the gut microbiota play a central role in IBD development, the modulation of intestinal bacteria may represent an active area of interest.

Despite the controversy related to the use of antibiotics and antimicrobial peptides in IBD, they can modify the composition of intestinal bacteria favoring the beneficial bacteria over those implicated in IBD pathogenesis [89]. Ciprofloxacin, aminoglycosides, or rifaximin have been reported to be effective against *Escherichia coli* and other Gram-negative enteric bacteria, and metronidazole for anaerobes (i.e., *Bacteroides fragilis*). Antibiotics can be useful in managing the primary phase of disease such as luminal and fistulizing CD and UC, or to counteract bacterial overgrowth, or septic complications [90].

Prebiotics, probiotics, postbiotics, and synbiotics can be helpful in the manipulation of the gut microbiota, also decreasing the epithelial cell apoptosis and intestinal mucosal inflammation [91]. 

Despite adequate therapy for IBD, pain may persist as one of the primary symptoms related to IBD, requiring the use of analgesics. Among the most common treatments, nonsteroidal anti-inflammatory drugs (NSAIDs), opiates, antidepressants, and anticonvulsants can be mentioned [92]. 

## 5. Drug Delivery Systems

To overcome the first-effect passage of conventional drugs and their rapid chemical or enzymatic degradation, the development of drug delivery systems (DDSs) could be a promising therapeutic strategy in IBD. They also highlight the advantage of prolonging the retention time of therapeutics in inflamed tissues and minimizing the administration frequency [93]. Few studies have been performed to develop new oral DDSs to target the colon affected by IBD. The choice of oral delivery is given by the major acceptance of this route by patients, and by the lowering related side effects. 

### 5.1. General Features of DDSs in the IBD

**Figure 2:**
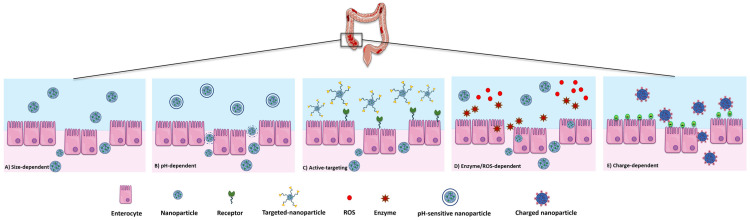
Different mechanisms of nanoparticle targeting.

The literature data suggest that DDSs have been designed considering the pathophysiological features of the inflamed intestinal tissue and exploiting specific mechanisms mediated features of DDSs such as size, charge, or pH [94] (Figure 2). 

Nanocarriers with small sizes, normally ranging from 10 to 200 nm, realize size-mediated targeting by taking advantage of the enhanced permeability and retention (EPR) effect [95]. In this case, the increased permeability of inflamed intestinal tissues, characterized by leaky vasculatures, facilitates the passive nanoparticle accumulation with the subsequent enhanced uptake by immune cells. This favorable size-mediated targeting on the GI tract has been demonstrated with polymeric nanoparticles based on polystyrene and poly-lactic-co-glycolic acid (PLGA) having sizes of 100 and 200 nm, respectively. They demonstrated, in an experimental model of UC, that orally administrated micro- and nanoparticles accumulate and penetrate the submucosal layer in a way strongly dependent on their size [96]. The drug targeting in the IBD is also largely influenced by the nanoparticle surface charges which allow useful electrostatic interactions between the DDSs and the inflamed intestinal environment, with a longer drug retention and bioavailability. The successful charge-mediated targeting was addressed with both cationic and anionic nanosystems. Indeed, cationic nanoparticles (NPs) may interact with the inflamed intestinal mucosa of patients with IBD, rich in negatively charged glycoproteins, while negative NPs can increase mucous interaction specifically binding to positively charged proteins [97]. For this purpose, Niebel et al. investigated polymethacrylate NPs in murine colitis models. The nanoparticles sized 120 nm and, since positively charged, were able to target the inflamed colonic mucosa via ionic interactions with the mucin with the subsequent decline of the myeloperoxidase activity. In other studies, chitosan-based drug delivery systems were used to adhere, exploiting their cationic surfaces, to the mucosal of the GI tract [98,99]. Regarding negatively charged formulations, Jubeh et al. demonstrated the capabilities of charged liposomes to interact with the inflamed intestinal mucosa is related to the superficial charge revealing a 2-fold higher adhesion capability for anionic liposomes [100]. The main trouble of these systems, related to the non-specific interactions that they can establish with other charged biological systems, may be partially overcome with more specific approaches. Ligand-mediated NPs may further increase the specificity and selectivity in GI delivery. In this case, surface-modified formulations emerged for their cell-targeting capabilities, favoring the controlled release of loaded drugs at the inflamed site of action and also minimalizing side effects related to unspecified targeting. This delivery strategy involves the conjugation, on the drug delivery surface, of selected ligands that target some specific receptors and cell adhesion molecules, such as folate receptor, mannose receptor, macrophage galactose lectin, transferrin receptor, glycoproteins including CD98, CD44 and F4/80, and the peptide transporter PepT1, overexpressed on the colonic epithelia and/or immune cells [101]. Other notable formulations are enzyme- and pH-responsive DDSs specifically designed to control the release of loaded drugs in response to specific environmental pHs or enzymes released by the colon microflora [102,103]. Gao et al. developed a colon-targeted system composed of a pH-sensitive polymer combination, including EL30D55 and ES100, PLGA as sustained release polymer, and Iridoid glycosides as a prototype of the loading molecule. This pH- and time-dependent system highlighted significant advantages due to its capability to improve drug bioavailability in the colon by promoting its accumulation and sustaining the release for a prolonged period [104]. Turanlı et al. also employed pH-responsive materials to develop formulations made of ES100 and ERL100, which, exploiting the external pH conditions, guarantee a triggered release of the loaded budesonide in the colon of patients affected by IBD [105].

### 5.2. Antibody-Loaded DDSs

Different antibody-loaded Nps or aptamers are reported in the literature for several applications [106]. Wang et al. investigated the oral delivery of infliximab, developing self-assembling nanoparticles composed of tannic acid and 1,2-distearoyl-sn-glycero-3-phosphoethanolamine-N-[methoxy(polyethylene glycol)-2000] (DSPE-PEG2k). Infliximab was released by Nps at the site of inflammation, proving in this way the capability of Nps to protect the antibody and to take it to the specific target site without premature degradation [107]. The delivery of infliximab was also proposed by Kim et al. using a ternary nanocomposite carrier which revealed, in a murine colitis model, its effectiveness in IBD treatment [108]. An example of redox-mediated targeting is reported by Li et al., who developed an oral nanocomplex consisting of a nano-core formed by infliximab, exploiting self-crosslinking technology with reactive oxygen species (ROS)-responsive cross-linkers. To improve the stability of antibodies to the acid environment and the target selectivity, hyaluronic acid was used to modify the external core due to the high expression of a specific receptor for hyaluronic acid on the surface of colon-inflamed cells. This nanocomplex overcame the decrease in the drug at the target site, its lower activity, and the risk of adverse events [109]. Ries et al. exploited polyester-based Nps as a surface on which immobilizing adalimumab and investigated their applicability in IBD in an experimental murine model of acute UC. The results showed that these DDSs lowered the enzymatic or chemical degradation of the antibody and the side effects, ameliorating its activity as anti-TNF-α [110]. Shrestha et al. also successfully encapsulate, for an oral administration, an anti-TNF-α mAb in PEGylated PLGA-based nanoparticles to counteract inflammation in an acute murine UC model as an alternative treatment conceived to increase blood circulation time and prevent undesirable interactions with biological tissues [111].

Based on these considerations, the therapeutic approach concerning this pathology has undergone a rapid evolution over the years. Currently, research has further moved from a traditional therapeutic approach towards more sophisticated systems such as biomaterials and organoids. Despite these many advances, supported by emerging therapies and technologies, still many challenges need to be addressed to confirm their effectiveness, feasibility, and safety since most of them are in research status hampering clinical use. Hopefully, in the future, organoids prepared using biomaterials will be effective in IBD management, mimicking intestinal conditions. 

Meanwhile, personalized therapy gained much interest. It may represent an opportunity but also a challenge for IBD since it requires a close interaction between doctors, health professionals, and patients. The doctors should individuate proper treatments in terms of drugs and doses considering the patient’s symptoms. Moreover, the treatments should be properly modulated depending on the response to the therapy. This approach based on active cooperation may represent a chance to get around the complexity of IBD.

## 6. Conclusions

Currently, there are several treatments for IBD, from conventional therapy to biologics. Conventional treatments represent the first-line therapy, especially in mild and moderate IBD. Due to the multifactorial pathogenesis of UC and CD, the advent of biologics has marked the introduction of new targets involved in the main inflammatory pathways, opening the way to novel treatments. TNF-α, integrin, and interleukin blockers have been approved since the 1990s. These drugs improved the quality of life, reducing hospitalization, surgery, and alleviating symptoms. Many studies regarding the discovery of alternative targets are ongoing. Monoclonal antibodies continue to play a significant role against IBD although in the future small molecules could regain importance in the pharmaceutical field. To offer novel perspectives on this disease, a deeper knowledge of the physiological and microbial changes in the GI tract may help for optimized IBD management. Among the most promising pharmaceutical strategies emerges the use of suitable formulations able to address the localized delivery of therapeutics to diseased tissues.

## Figures and Tables

**Table 1:** Traditional drugs used for IBD: administration routes and mechanism of action.

Traditional Drugs	Class of Drugs	Administration Route	Mechanism of Action	Refs.
Sulfasalazine	Aminosalicylates	Oral	The inhibition of IL-1, TNF-α production, NF-kB, and lipoxygenase pathways	[21,22,23,24,25,26]
Prednisone	Corticosteroids first generation	Oral or parenteral	The inhibition of the immune system	[29]
Beclomethasone dipropionate	Corticosteroids second generation	Oral or topical	The inhibition of the immune system	[30,31]
Azathioprine	Thiopurines	Oral	The inhibition of T lymphocyte proliferation and activation	[32]
Methotrexate	Antimetabolites	Parenteral	Decreasing pro-inflammatory cytokine production and leading to the apoptosis of T cells	[33,34]
Cyclosporine	Immunosuppressives	Oral or intravenous	Calcineurin inhibition	[35]
Tacrolimus	Immunosuppressives	Oral or intravenous	Calcineurin inhibition	[36,37]
